# Time-dependent impact of co-matured manure with elemental sulfur and biochar on the soil agro-ecological properties and plant biomass

**DOI:** 10.1038/s41598-023-31348-7

**Published:** 2023-03-15

**Authors:** Jiri Holatko, Tereza Hammerschmiedt, Adnan Mustafa, Antonin Kintl, Petr Skarpa, Pavel Ryant, Tivadar Baltazar, Ondrej Malicek, Oldrich Latal, Martin Brtnicky

**Affiliations:** 1 Department of Agrochemistry, Soil Science, Microbiology and Plant Nutrition, Faculty of AgriSciences, Mendel University in Brno, Zemedelska 1, 61300 Brno, Czech Republic; 2 Agrovyzkum Rapotin, Ltd., Vyzkumniku 267, 788 13 Rapotin, Czech Republic; 3 Faculty of Chemistry, Institute of Chemistry and Technology of Environmental Protection, Brno University of Technology, Purkynova 118, 612 00 Brno, Czech Republic; 4 Faculty of Science, Institute for Environmental Studies, Charles University in Prague, Benatska 2, 12800 Prague, Czech Republic; 5 Agricultural Research, Ltd., Zahradni 400/1, 664 41 Troubsko, Czech Republic

**Keywords:** Microbiology, Environmental sciences

## Abstract

Farmyard manure is the most common type of organic fertilizer, and its properties depend mainly on the type of livestock, bedding material and the conditions of fermentation. Co-maturing of manure with other amendments to modify its final properties has been seen as a win–win strategy recently. This study aimed to evaluate the differences in the effect of unenriched manure and manures co-matured with biochar, elemental sulfur or both amendments on the soil physico-chemical and biological properties, and plant (barley, maize) biomass production. For this purpose a pot experiment was carried out in a time-dependent way. Samples were taken from 12 week-lasting (test crop barley) and 24 week-lasting (test crop maize) pot cultivation carried out in a growth chamber. Co-matured manure with biochar showed the highest rate of maturation expressed as humic to fulvic acid ratio, its amendment to soil significantly increased the dry aboveground biomass weight in the half-time (12 weeks) of experiment. However, the effect vanished after 24 weeks. We received for this variant highest long-term (24 weeks) contents of total carbon and nitrogen in soil. Contrarily, co-matured manure with biochar and elemental sulfur led to short-term carbon sequestration (the highest total carbon in 12 weeks) due to presumed retardation of microbial-mediated transformation of nutrients. We conclude that the prolonged pot experiment with biochar or elemental sulfur enriched manure led to the increased recalcitrancy of soil organic matter and retardation of soil nutrient transformation to the plant-available form.

## Introduction

One of the main negative consequences of poor soil management, unsustainable agricultural practices, pollution, wind and water erosion is the loss of soil organic matter (SOM)^1–3^. That is closely related to decrease in soil fertility and decline in biological activity and quality of soil. Application of organic fertilizers can restore and maintain a suitable SOM content in soil^4^. Farmyard manure is the most common type of organic fertilizer with key role in maintaining of quality and healthy arable soil in sustainable agriculture^5^. Manure amendment to the agricultural soil positively affects formation of SOM, improves the soil structure as well as the fertility of the soil^6,7^. Moreover, manure contributes significantly to increasing soil water storage, crop yield and soil microbial activity^8–10^. It represents a good source of nutrients, especially carbon, nitrogen, phosphorus, and other minerals for both, plants and soil organisms, including microbes^11^. The properties of manure are variable and depend mainly on the type of livestock, bedding material and the conditions of fermentation, which can be modified to achieve the intended quality of the product^12^.

However, co-composting is another way how to modify the properties of final manure. Co-composting is based on the affecting the processes and manure quality via amendment of another mostly organic materials^13^. For example, biochar has a great potential for enhancing composting of manure, it modifies the thermodynamics and heat generation in fermentation process^14^, improve compost mixture physicochemical properties^15^, enhance microbial activities^16^, promoted organic matter decomposition^17^, reduced greenhouse gas emissions^18^, namely ammonia^19^ upgrade compost quality via increased total/available nutrient content^20^, enhanced maturity, and decreased phytotoxicity and nutrient leaching^21^. The time-related effect of biochar-based organic amendments is another issue which has been in the focus of several studies^22–24^. The primary effect of biochar on the soil properties was evaluated and reported by several studies^25–28^ as well as long-time effect^22,29,30^, but the research focused on the comparison and contrasting the time-shifted impact on soil^23^ might be broaden.

Amendment of manure with elemental sulfur have also shown promising results in previous studies. Application of elemental sulfur incerases nutrient availability^31,32^, namely in calcareous soils^33,34^, and serves as an additive changing the physico-chemical properties of soil^35,36^. Moreover, it has a potential to increase crop yields^37^. Europe's agricultural faces recently an emerge of sulfur deficiency due to decline in SO_2_ emissions, which has been reduced to 20% compared to levels 30 years ago^38^. The general data of soil analyses carried out in the Czech Republic show that 85% of soil has low sulfur content^39^. Nevertheless, the effect of elemental sulfur on manure quality, soil properties and plant growth upon the combined treatments with manure or biochar have been reported in few studies^40–42^, and still raises a need for further investigation.

The objectives of this study were to evaluate: (I) the differences in the effect of unenriched manure and manures co-matured with amendments (biochar, elemental sulfur, and combination of both) on the soil physico-chemical and biological properties, and plant biomass production, (II) the time-effect of soil amendment with these various manure types on the respective properties and potential changes in the impact in a shorter time (12 weeks) and longer time (24 weeks) of pot experiment. It was hypothesized that early effects of amendments would be more significant and may become weakened and less demonstrable with longer period of interaction with soil. A presumption was adopted that sulfur and/or biochar enhanced the growth and activity of manure-derived microflora which would accelerate the nutrient transformation processes in the amended soil and expedited changes in soil chemistry would culminate in the early, not in the later phase of the whole trial.

## Materials and methods

### Production and analyses of co-matured manures

Farmyard manure was collected from cattle breeding farm without marketable milk production at Research institute for cattle breeding Ltd., located in village Rapotin, Czech Republic, Central Europe (49°58′46.4″ N, 17°0′26,6″ E). The basic physical and chemical properties of manure before activation (via co-maturation) were as follows: pH (H_2_O) 9.035 ± 0.298, N_kjeldahl_ 2.578 ± 0.085 g kg^−1^, C_tot_ 9.100 ± 0.300%, HA:FA 0.565 ± 0.019, S_tot_ 0.786 ± 0.026 g kg^−1^, C_tot_ : S_tot_ 11.578 ± 0.039, dsr 10⋅10^6^ ± 0.01⋅10^6^ copies⋅g^−1^. Manures were prepared in these variants: unenriched manure (M), manure mixed with elemental sulfur (M + S), manure mixed with biochar (M + B), manure mixed with biochar and elemental sulfur (M + B + S), more detailed description of the variants in (Table 1).

**Table 1:** Preparation of manure variants—dosage of amendments, moisture content.

Variant	Manure per barrel	Biochar (B) or elem. sulfur (S) per barrel	Dry matter ratio M to BC and/or to S	Moisture content of the final manure
M	10 kg	0	0	70.0%
M + S	10 kg	0.14 kg	2.5:0.09	68.2%
M + B	10 kg	4.0 kg	2.5:2	64.0%
M + B + S	10 kg	4.0 kg 0.14 kg	2.5:2:0.09	57.6%

Biochar used in the experiment was commercially produced from agricultural waste (cereal husks, sunflower peels and fruit mud) at 600 °C (Sonnenerde GmbH, Riedlingsdorf, Austria). According to manufacturer, the properties of the biochar were as follows: elements (g·kg^−1^) C 866, N 3.0, O 10.0, H 14.2, P 2.5; Ash_550 °C_ 11.7%, salts 0.42%, pH (CaCl_2_) 8.5, BET surface area 288.5 m^2^⋅g^−1^, bulk density 120 kg⋅m^3^. Elemental sulfur was produced as waste product during desulfurization of biogas in THIOPAQ scrubber (Paques, Netherlands).

Manure and amendments were dosed into the 50-L tightly closable barrels according to (Table 1), thoroughly kneaded and mixed. For a further manure application to the soil equivalent to 50 t ha^−1^, doses equal to 20 t ha^−1^ of biochar and 0.7 t ha^−1^ of elemental sulfur were calculated. Each variant was prepared in 3 replicates and the mixtures tightly closed to decrease desiccation and prevent air contamination from the surroundings. Activation process was similar as reported previously^43^, carried out for 8 weeks under room temperature (25 ± 2.5 °C) and aerobic conditions. Temperature and relative air humidity were monitored (Table 2) every 2 weeks and the content of each barrel was mixed.

**Table 2:** Relative air humidity (ϕ) and temperature (T) of manure variants during maturation.

M			M + S		M + B		M + B + S	
week	ϕ [%]	T [°C]	ϕ [%]	T [°C]	ϕ [%]	T [°C]	ϕ [%]	T [°C]
2	72.0	25.4	68.0	24.7	73.0	24.6	75.0	24.4
4	68.0	25.2	76.0	23.6	77.0	24.2	76.0	24.2
6	70.0	25.1	71.0	24.8	71.0	24.9	69.0	24.1
8	70.0	24.8	73.0	22.6	72.0	22.7	72.0	22.5

Manure pH in H_2_O was determined by electrolytic measurement according to ISO 10,390:2005^44^, total Kjeldahl N (N_Kjeldahl_) was determined by Kjeldahl procedure according to ISO 11,261:1995^45^, total carbon (C_tot_) was measured by dry combustion according to ISO 10,694:1995^46^, humic and fulvic acids were determined in the extract according to ISO 19,822:2018^47^, S_tot_ was determined by ICP-OES according to EN 15,749^48^, sulfur-reducing microorganisms (*dsr*) were determined by real-time qPCR detection of gene coding for sulfur reductase according to^49^. The determined parameters for manure amendments are listed in (Table 3).

**Table 3:** Chemical properties of the prepared matured amendments at the end of maturation.

Variant	pH (H_2_O)	N_Kjeldahl_ [g kg^−1^]	C_tot_ [%]	HA:FA	S_tot_ [g kg^−1^]	C_tot_: S_tot_	dsr [Copies g^−1^]
M	9.04 ± 0.01	24.79 ± 0.46	9.1 ± 0.12	0.79 ± 0.01	7.86 ± 0.38	11.58	10.3 10^6^ ± 1.0 10^6^
M + S	4.85 ± 0.01	23.93 ± 0.58	9.99 ± 0.91	0.77 ± 0.03	15.07 ± 2.00	6.63	139.2 10^6^ ± 17.0 10^6^
M + B	8.71 ± 0.01	19.91 ± 0.74	21.01 ± 0.34	2.04 ± 0.18	3.27 ± 0.11	64.27	9.6 10^6^ ± 0.9 10^6^
M + B + S	5.71 ± 0.01	18.78 ± 0.61	24.09 ± 0.86	1.18 ± 0.03	11.60 ± 0.54	20.77	73.3 10^6^ ± 8.3 10^6^

### Collection of soil, treatment plan and pot experiment

The experimental soil was collected from the rural area near the town Troubsko, Czech Republic (49°10′28"N 16°29′32"E). The soil was a silty clay loam according to USDA Textural Triangle, Haplic Luvisol according to WRB soil classification (FAO, 2014) same as in the previous study^50^, content of nutrients in g·kg^−1^: total carbon (TC) 14.00, total nitrogen (TN) 1.60, P 0.10, S 0.15, Ca 3.26, Mg 0.24, K 0.23, pH (CaCl_2_) 7.29.The topsoil was dug up to the depth of 15 cm, big parts (stones, lumps) were removed on a sieve (2 mm). The sieved soil was mixed with fine quartz sand (0.1–1.0 mm) in weight ratio 1:1.

The three tested variants were prepared by thorough mixing of 5 kg of experimental soil with 200 g of particular manure type per a pot, equals to 50 t⋅ha^−1^. Unamended control (control) contained only 5 kg of experimental soil. Each variant was prepared in four pots of volume 5 L and marked equally to the used manure type.

To investigate the effectiveness of prepared variants, barley (*Hordeum vulgare* L.), a crop with lower requirements of sulfur, was grown for first 12 weeks of the experiment. Each pot was sown with 16 barley seeds 2 mm under soil surface. All pots were placed randomly into growth chamber (CLF Plant Climatics GmbH, Germany) and rotated every other day to ensure homogeneity of conditions for the treatments. Controlled conditions were set as follows: 12 h long photoperiod, light intensity 370 µmol·m^−2^·s^−1^, temperature (day/ night) 20/12 °C, relative air humidity (day/ night) 45/70%. Pots were watered with distilled water. Soil moisture was determined gravimetrically and maintained at 65% of water holding capacity throughout the experiment^51^. The number of plants were reduced to 12 in each pot after 2 weeks. After 12 weeks, the aboveground biomass of barley plants was harvested, and 100 g of soil was taken from each pot for analyses.

Then, each pot with remaining soil and barley roots was sown with five maize (*Zea mays* L) seeds, for maize is a crop with higher demand to sulfur content in soil. Seedlings were reduced to 2 plants after 2 weeks. The experiment was carried out under the same growth conditions for further 12 weeks, i.e., 24 weeks in total, following same sampling protocols as described above.

### Plant biomass and soil properties determination

Harvested barley and maize biomass were dried at 60 °C to constant weight. The dry above ground biomass (AGB) was determined gravimetrically using the analytical scales.

The soil samples were homogenized by sieving through a 2 mm mesh. Air-dried samples were used for determination of soil pH in CaCl_2_^43^, total soil carbon (TC) and nitrogen (TN) content^45,52^. The samples stored at 4 °C were used for determination of soil basal (BR) and substrate induced respirations: D-glucose (Glc-SIR), D-trehalose (Tre-SIR), N-acetyl-β-D-glucosamine (NAG-SIR), L-alanine (Ala-SIR), L-lysine (Lys-SIR), L-arginine (Arg-SIR), according to^53^. The freeze-dried samples were prepared for the enzymes, arylsulfatase (ARS), N-acetyl-β-D-glucosaminidase (NAG), and urease (Ure), activity assays:^54^.

### Statistical analyses

Data obtained from the performed measurements were statistically analyzed using the principal component analysis (PCA), one-way analysis of variance (ANOVA), Tukey HSD post-hoc test (at significance level *p* = 0.05), and Pearson correlation analysis (Program R, version 3.6.1)^55^.

The Rohlf’s PCA Analysis was used to evaluate the mutual dependence among the properties and their values in individual compared variants of amended soil. The results of Pearson’s correlation analysis were mentioned when value of the correlation coefficient r was: 0.5 < r < 0.7 (moderate correlation) and 0.7 < r < 0.9 (high correlation)^56^.

## Results

### Plant traits under applied amendments

**Figure 1:**
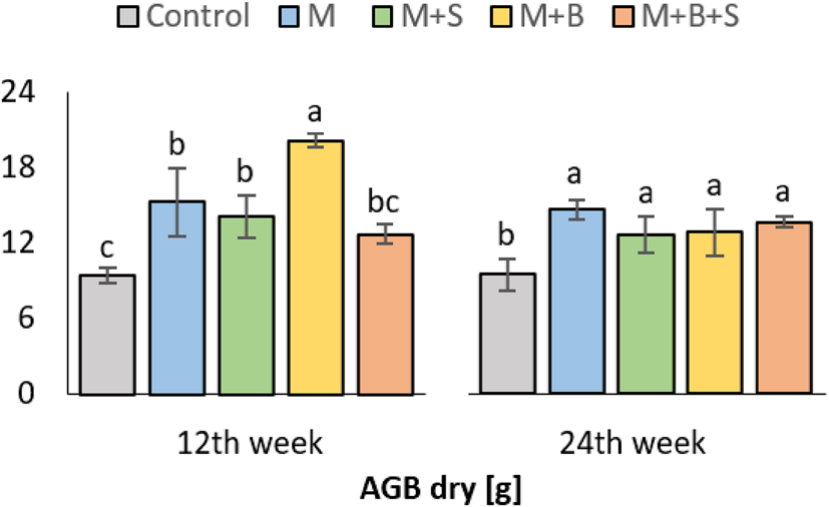
Dry aboveground biomass of barley under (12th week) and maize under (24th weeks) duration. *Average values (n* = *4) are displayed, error bars are standard error of mean. The different letters of variant values indicate a statistical difference among them at the level p* ≤ *0.05.*

After 12 weeks of cultivation, the significantly highest weight of dry aboveground biomass (AGB dry) of barley was found in the variant M + B, but M and M + S variants also exerted AGB dry significantly increased as compared to the unamended control, (Fig. 1). Significant (*p* ≤ 0.001; *p* ≤ 0.01) moderate positive correlation (Fig. S2a) was observed between AGB dry and N-acetyl-b-D-glucosamine-induced, D-glucose-induced, and L-lysine-induced respiration (NAG-SIR, Glc-SIR, Lys-SIR; r was 0.77, 0.69, 0.67, respectively). At the end of experiment (24 weeks), there was no statistical difference among all amended soil variants, which exerted significantly increased AGB dry of maize in comparison to the control, (Fig. 1). The final values of AGB dry significantly (*p* ≤ 0.001) and moderately positively correlated with soil total carbon (TC, r = 0.64) and highly negatively correlated with pH (r =− 0.72), (Fig. S2b).

### Soil physico-chemical properties

**Figure 2:**
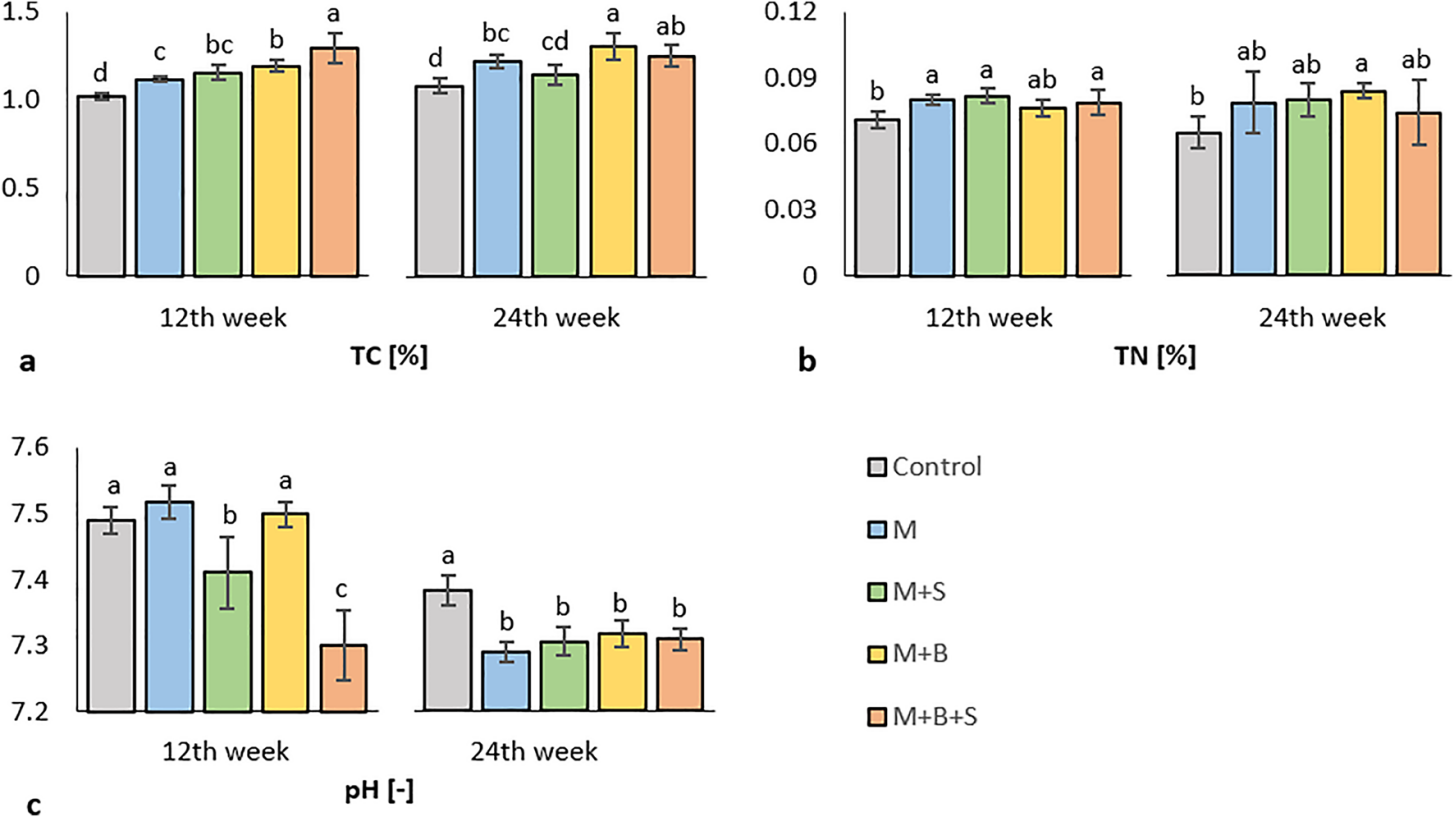
Soil total carbon (**a**) and nitrogen (**b**) content and pH (CaCl_2_) (**c**) after 12 and 24 weeks of cultivation*. Average values (n = 4) are displayed, error bars are standard error of mean. The different letters of variant values indicate a statistical difference among them at the level p* ≤ *0.05.*

After 12 weeks of cultivation, soil pH of was significantly decreased (as compared to the control and variants M and M + B) in both variants amended with elemental sulfur, M + S and M + B + S. Moreover, the M + B + S variant showed significantly lowest pH, (Fig. 2). The pH after 12 weeks correlated (Fig. S2a) significantly (*p* ≤ 0.001) and moderately negatively with TC (r = − 0.67) content. However, this assumed sulfur-derived effect seemed short-timed again, thus pH after 24 weeks of cultivation exerted no significant difference among all amended variants, which all were significantly decreased compared to the control, (Fig. 2). The pH after 24 weeks negatively correlated (Fig. S2b) with TC less strongly albeit still apparently (r = − 0.53, *p* ≤ 0.001). The overall effect of manure on pH was more significant than the impact of any amendment in the end of experiment.

**Figure 3:**
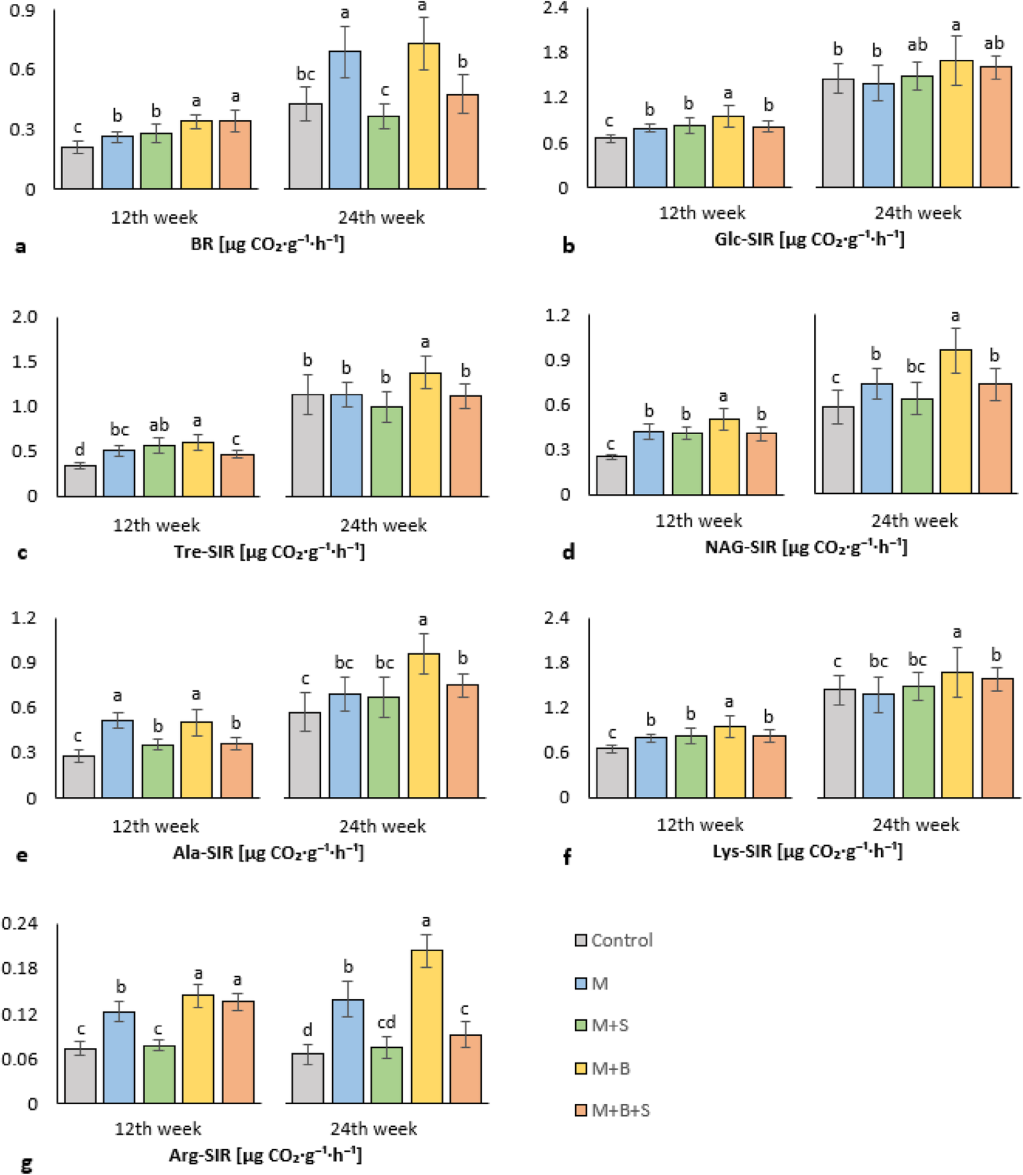
Soil basal (**a**) and D-glucose-induced (**b**) D-trehalose (**c**) and N-acetyl-b-D-glucosamine (**d**) induced respiration, L-alanine (**e**), L-lysine (**f**) and L-arginine (**g**) induced respiration after 12 and 24 weeks of cultivation*. Average values (n* = *4) are displayed, error bars are standard error of mean. The different letters of variant values indicate a statistical difference among them at the level p* ≤ *0.05.*

After 12 weeks of cultivation, the total soil carbon (TC) was significantly the highest in the M + B + S and variant M + B contained significantly more TC compared to the variant M + S, M and control, Fig. 2a. These results correspond to the total carbon content in the applied manure variants, which were (in average): 24.1% (M + B + S), 21.0% (M + B), 10.0% (M + S), 9.0% (M). A putative contribution of sulfur addition to the carbon sequestration was assumed, however, only increase in biochar-derived carbon was significantly detectable. The higher TC availability likely anticipated higher carbon utilization, as far as we can assume from the moderate positive correlation of TC and respirations (e.g., BR, NAG-SIR, Arg-SIR; r was 0.61, 0.62, 0.64, respectively; *p* ≤ 0.001); furthermore, TC correlated with TN (r = 0.55; *p* ≤ 0.001). Again, vastly different was the determination of TC at the end of experiment (24 weeks), when the highest value was detected in M + B variant. TC values of M + B and M + B + S variant were significantly higher compared to the variants M + S and control, Fig. 3a. At the end of experiment, TC correlated (Fig. S2b) most with total soil nitrogen (TN, r = 0.65) and with AGB dry (r = 0.64), which corroborated the presumed mutual relation of nutrient content (or sequestration) and high plant (maize) biomass yield. Higher TC content was again a putative prerequisite for higher transformation activity, as indicated by TC and respiration correlation (i.a. Arg-SIR, NAG-SIR, Ala-SIR; r was 0.65, 0.58, 0.53, respectively; *p* ≤ 0.001).

In the early phase of experiment, the soil TN was significantly increased in variants M, M + S, and M + B + S as compared to the control, Fig. 2b. These results correspond (with exception of co-matured manure M + B) to the values of TN content in the respective manure variants: M (2.48%), M + S (2.39%), M + B + S (1.88%), M + B (1.99%). The presumed role of enhanced nutrient oxidation in mitigation of nitrogen volatilization was ascribed from the significant positive moderate correlation between TN and N-acetyl-b-D-glucosamine-induced respiration (NAG-SIR, r = 0.61), Fig. S2a. The final TN values of amended variants after 24 weeks exerted no significant increase compared to the control, except of the statistically highest TN in the M + B variant, Fig. 2b. The only detected markable positive correlation was between TN and TC (r = 0.65), (Fig. S2b).

### Soil microbial activity

Microbial activity in soil was determined as soil respiration (basal and induced by various substrates) and activity of soil enzymes. In the first half of the experiment (12 weeks) the significantly increased basal respiration (BR), compared to the control, was received in all manure-amended variants. However, the BR values of variants M + B and M + B + S were significantly higher in comparison to the M and M + S variants, Fig. 3a. The BR correlated (Fig. S2a) significantly (*p* ≤ 0.001), positively and moderately with TC (r = 0.61), and with all respiration types, the most with NAG-SIR (r = 0.65), Lys-SIR and Arg-SIR (r was 0.68 both). At the end of experiment (24 weeks), the previously enhanced BR in the variant M + B + S was mitigated to values comparable to the control, the significantly increased BR was observed in the M and M + B soil, (Fig. 3a). The data from the 24th week of cultivation showed also a significant positive moderate (to high) correlation between BR and NAG-SIR, Lys-SIR, Arg-SIR (r was 0.67, 0.65, 0.78, respectively), (Fig. S2b). Very similar results as the basal respiration showed almost all types of substrate-induced respiration.

The M + B variant showed the significantly highest Glc-SIR, Tre-SIR, NAG-SIR and Lys-SIR compared to all other variants after 12 weeks of barley cultivation. The control exerted significantly decreased Glc-SIR, Tre-SIR, NAG-SIR, Ala-SIR and Lys-SIR compared to the variants amended with any manure type, (Fig. 3a-g),. After 24 weeks, the significantly highest values of all substrate-induced respirations were found in the M + B. Nevertheless, the control variant exerted no significant difference to the M, M + S, and M + B + S variants in parameters Glc-SIR, and Tre-SIR, (Fig. 3b). After 12 weeks, Glc-SIR significantly (*p* ≤ 0.001) positively correlated with all respiration types—the most with Tre-SIR, NAG-SIR (r were 0.72 and 0.73, respectively)—and after 24 weeks with NAG-SIR, Ala-SIR, Lys-SIR (r were 0.51, 0.65, 0.72, respectively), (Fig. S2a, b).

After 12 weeks, significant positive correlation was found between Lys-SIR (Arg-SIR) and AGB dry (r was 0.65 and 0.67, respectively), Ala-SIR correlated (*p* ≤ 0.001) positively with all respiration types (e.g., Lys-SIR and Arg-SIR; r was 0.59 and 0.60, respectively) and with Ure (r = 0.46), whereas negatively with arylsulfatase (ARS, r = 0.50) (Fig. S2a). At the end of experiment, the mutual correlations of Ala-SIR with both other respiration types (e.g., Lys-SIR and Arg-SIR, r was 0.88 and 0.72, respectively) and Ure (r = 0.46) were again revealed, even higher than in the early cultivation. Whereas the relation to TC in soil was more apparent: TC and Ala-SIR, Arg-SIR showed r value 0.53 and 0.65, respectively, (Fig. S2b).

**Figure 4:**
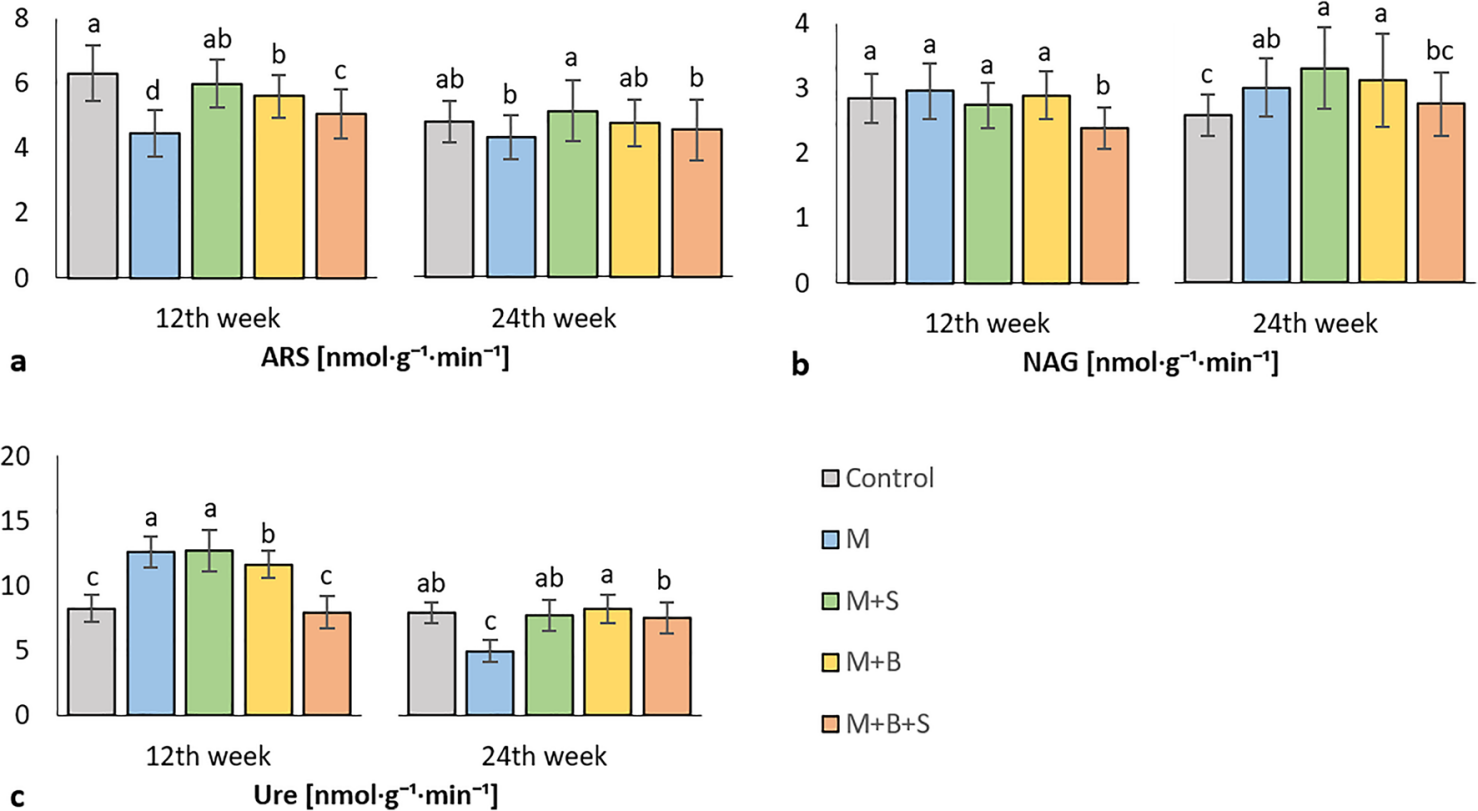
Soil arylsulfatase (**a**), N-acetyl-b-D-glucosaminidase (**b**), and urease (**c**) activity after 12 and 24 weeks of cultivation. *Average values (n* = *4) are displayed, error bars are standard error of mean. The different letters of variant values indicate a statistical difference among them at the level p* ≤ *0.05.*

After 12 weeks of experiment, the highest average soil ARS activity was received in the control variant, which showed comparable value to M + S variant and significantly increased ARS compared to the variants M, M + B, M + B + S, Fig. 4a. The PCA biplot showed that ARS was antagonistic to the BR in soil, (Fig. S1a). The end-values (24th week) of ARS were comparable among the control and all other variants, from which M + S had a significantly increased ARS in comparison to the variants M and M + B + S (Fig. 4a).

In the early phase of the experiment (12 weeks), NAG activity did not differ significantly among all variants except of M + B + S, which was significantly decreased compared to the others (Fig. 4b). The long-termed cultivation (24 weeks) preserved the low NAG activity in M + B + S, which only was comparable to the control. The other variants (M, M + B, M + S) exerted significantly increased NAG compared to the control (Fig. 4b). The PCA biplot revealed synergism between NAG and Lys-SIR, Ala-SIR, (Fig. S1b).

Urease (Ure) activity was already presented as the significantly highest in the M and M + S variants, but also M + B variant was significantly increased compared to the control after 12 weeks, (Fig. 4c). A significant positive correlation (Fig. S2a) was received between Ure and respirations (Tre-SIR, NAG-SIR, Ala-SIR; r were 0.5, 0.39, 0.46; *p* ≤ 0.001), enzymes (Phos, GLU; r were 0.57, 0.33; *p* ≤ 0.001) as well as AGB dry (r = 0.52). The end-values (after 24 weeks of the experiment) of Ure in control and variants with enriched manure (M + B, M + S, M + B + S) were significantly increased as compared to control variant, the highest average value of Ure activity showed M + B variant (Fig. 4c).

## Discussion

### Plant traits under applied amendments

In the early phase of experiment, only the co-matured manure with biochar (M + B) had a significantly higher beneficial effect on plant (barley) biomass yield in comparison to the application of unenriched manure (M), thus it increased the barley AGB dry. It was in the line with the reported results^55^. Nevertheless, the observed biochar-derived benefit cannot be coupled with the increased soil water holding capacity. The other types of enriched co-matured manures (M + B + S, M + S) increased AGB dry compared to the control soil variant, but not compared to the unenriched manure (M). We presumed the beneficial effect of elemental sulfur supplement to the soil used in this experiment, because the chosen soil type had relatively low total S content, compared to other arable soils in the Czech Republic^56^. The M + B manure showed the highest rate of maturation determined as humic:fulvic acid ratio—HA:FA = 2.04 (Table 3). These results agreed with the referred enrichment of manure humic acids in relation to the fulvic acid fraction in manure–biochar mixture^57^. Putatively, these properties, together with high amount of pyrolyzed carbon matter, anticipated the most demonstrable contribution to soil nutrient availability and plant biomass yield. Nevertheless, it was assumed (similarly as in^57^) that manure–biochar mixture featured high polymerization degree of humic-like substances at the end of the maturation process, therefore amendment of soil with this respective manure (+ biochar) reduced in the later phase of the experiment (12th to 24th week) the degradability of total soil (mainly organic) carbon. It presumably resulted from the relative enrichment of recalcitrant organic matter due to the preferential degradation of labile, non-aromatic organic matter, similarly as referred by^58^. Contrarily, the co-matured manure with combined biochar and elemental sulfur was found the least beneficial to the soil fertility and barley biomass, putatively due to the adverse co-effect of the lowest pH, nutrient transformation rate indicated by the lowest values of Ure, Phos, NAG, and the highest C:N ratio. These features documented assumed retarded mineralization and mediation of nutrients acquisition by plants.

Nevertheless, the differences derived by application of variable manures were mitigated in the second half of the experiment, leading to the comparable maize yield in all manure-amended soils. Although, the M + B variant revealed the highest average TC, TN, Ure activity indicating increased nitrification rate, the M variant exerted the highest average AGB dry (Fig. 1). We assume that the prolonged incubation of biochar under experimental conditions led to the increased recalcitrancy of soil organic matter (SOM) and retardation of nutrient transformation to the plant-available form. However, the results could also be affected by the sensitivity of barley to soil pH and the plasticity of maize to the experimental conditions.

### Soil physico-chemical properties

The significant decrease of pH value in the M + S variant was assumed and explainable as there are studies which referred to the acidifying effect of elemental sulfur on the pH of organic fertilizers^42,59^ or soil^60^. The novel finding is that combination of biochar and elemental sulfur in co-matured manure even more decreased soil pH after short period (12 weeks). That presume an expected specific impact of elemental sulfur amendment and its transformation on the biochar interaction and decomposition in soil, which differed from sole biochar interaction with manure and its impact on soil pH value. We explained this observation by the putatively increased oxidation of biochar in the M + B + S manure and soil due to the parallel oxidation of elemental sulfur. It was in the line with study^61^ which expected microbial induced "in situ digestion" of biochar by oxidation of elemental sulfur. This biochar digestion could subsequently reduce its pH-buffering capacity and lead to more significant pH decrease. After 24 weeks of experiment, this significant effect faded out due to the strong negative impact of all manure-based amendments (M, M + B, M + B + S, M + S) on the soil pH. Such observation is rare, thus most authors referred to pH-increasing effect of either manure^62^ of biochar^63^ soil amendment. The study^64^ reported of the pH-decreasing effect of biochar due to the adsorption of ammonium nitrogen on the biochar surface. We assume that its stabilization via biochar in M + B, M + B + S variants or decrease in ammonium concentrations via its consumption/conversion in M, M + S variants caused the observed change in pH.

The highest contribution of M + B + S manure amendment to soil TC was related to the highest carbon content in the end of manure maturation, (Table 3). It was in the agreement with the reported sulfur-enriched biochar positive effect on carbon sequestration^65^. The contribution of sole biochar in co-matured manure to the total soil carbon is well known^66^, less documented is the positive effect of co-matured manure with sulfur on soil organic matter, fertility, and crop growth^67^. However, the strong positive effect of elemental sulfur to TC sequestration was only short-termed, while the stabilizing effect on TC of the co-matured manure with sole biochar was more significantly lasting. These finding are related to references of short-termed beneficial effect of sulfur-enhanced biochar^68^, whereas storing carbon in soil due to the amendment of biochar was multiply evaluated as long-termed^69^.

Based on the previous reports^42^, we assumed mitigation of nitrogen (N) loss via volatilization due to higher oxidation of reduced N forms in the early phase of the experiment (12 weeks). The variants with two highest values of both, total soil and total manure nitrogen, showed the highest manure nitrate content: M + S (31.21 mg⋅kg^−1^) and M (14.64 mg⋅kg^−1^) too (Table 3). We claim that the reason was the enhanced nitrogen conversion rate compared to the putative biochar-mediated retardation, documented i.e., by positive relation between TN and NAG-SIR. The mitigation of this early-phase induced-effect of manure amendment on soil TN was observed again at the end of experiment. The only significant increase of TN content in the M + B variant repeatedly corroborated assumption that the general enhancement of nutrient sequestration by the manure + biochar amendment has the long-termed consequence.

### Soil microbial activity

Soil respiration (basal and substrate-induced) is an indicator of catabolic activity of aerobic microbiota in soil^70^, it monitors the carbon mineralization and functional diversity in soil microbial community. In the first (12 weeks) and second (24 weeks) half of the experiment, the highest basal and induced by 3 types of carbohydrates respiration was found in M + B variant. Basal respiration was significantly increased in M + B + S variant (compared to the control) too, however induction with D-glucose, D-trehalose, and N-acetyl-β-D-glucosamine showed that the respiration potential of M + B + S soil is lower than of M + B. Elemental sulfur significantly weakened enhancing effect of biochar in manure on soil respiration. Thus, the strongest respiration-stimulatory effect of co-matured manure + biochar amendment on the soil was detectable during the whole experiment. We explained this observation by very high content of carbonaceous organic matter in the respective manure type M + B, TC (21.01%) and concurrently its highest maturity indicated by HA:FA (2.04%). It was reported that the level of manure maturity is crucial for the improvement of soil fertility^71^ and the significant positive effect of manure^72^ and biochar^73^ on the soil respiration was reported too. The other manure types (M, M + S) with TC (9.10%, 9.99%) content lower than M + B manure (TC 21.01%) either did not contribute so significantly to the soil oxidizable carbon pool or could stimulate also anaerobic respiration which decreased the rate of CO_2_ emission. These general features of soil respiration in soil variants affected putatively also particular types of SIR (NAG-SIR, Lys-SIR, Arg-SIR), which correlated (Fig. S2a, b) significantly positively with TC, the relevant marker to the available catabolizable carbon compounds in soil.

The results from determination of amino acid (AA: Ala, Lys, Arg)-induced respiration in the M + B soil shortly affected (12 weeks) by co-matured manure and biochar variant showed the increased (highest) values compared to the control. Farmyard manure was referred to increase total soil amino acids as well as soil oligopeptide immobilization^74^. Biochar induced elevated lysine content in rhizosphere too^75^. Stimulation of catabolism and carbon mineralization was putatively joined with enhanced nutrient transformation, leading to higher availability and acquisition by plants, subsequently indicated by correlating increased AGB dry (Fig. S2a). Amendments of other types of manure also stimulated soil microbiome for enhanced Ala- and Lys-SIR after 12 weeks of experiment, but the positive effect of these variants vanished at the end of experiment. Except of M + B, at the end of experiment only variant M + B + S putatively sequestered sufficient amount of organic (nitrous) matter for AA-induced respiration significantly increased in comparison to the control. Similar observation was previously reported to crop yield correlated (Fig. S2b) positively with the ratio of soil respiration to SOM^76^. The AAs-SIR showed higher correlation with TC in the samples taken at the end of the experiment than after 12 weeks. Moreover, the highest values of respiration in M + B variant were related to the highest soil TC value, which was likely biochar-mediated. On the contrary, a negative correlation between Ala-SIR and ARS after 12 weeks of experiment may explain the presumed negative effect of sulfur mineralization (in the M + S soil) on the soil respiration. This novel assumption was in contrast with up-to-now reported joint increase in both, soil respiration and arylsulfatase^77^.

The results of ARS determination in the 12 week-incubated soil samples revealed the highest activity in the control variant. We explained this finding by the lowest content of plant available sulfur in the control soil, because this variant was not supplied with any source of sulphates. Therefore, the plant and microbial demand for sulfur was coupled with its higher mineralization rate, leading to the ARS induction. The positive relation between microbial biomass sulfur and ARS was reported^78^. The study by^79^ referred to decreased sulfur mineralization upon additions of carbon or sulfur in the organic amendment. Further, ARS was enhanced in the M + S variant, assuming that the higher access of elemental S in the respective manure without any limitation due to the biochar stabilization led to the significantly increased sulfur turnover and subsequently to induction in ARS. The presumption that ARS activity is tightly negatively related to the mineralization rate in soil, is corroborated by the antagonism with BR in soil. The later phase of the experiment was characterized by increased carbon mineralization rate, BR values and plant sulfur uptake, which led to ARS decrease in the most of soil variants.

N-acetyl-β-D-glucosaminidase (NAG) is an indicator of nitrogen mineralization and fungal biomass content and its turnover in soil^80^. We considered adverse effects of elemental sulfur on the fungal growth and soil biomass (and concurrently also NAG activity) in this experiment, in the line with reports by^81^. However, subsequently we presumed a proceed in the sulfur transformation and a mitigation of the putative fungistatic effect of elemental S, and we indeed evidenced the highest increase in NAG activity in the variants M + B and M + S, which exerted high TN content and high respiration potential as well (determined as Glc-SIR).

Urease (Ure) is a key and ubiquitous enzyme in the soil nitrogen mineralization^82^. Thus, the early experimental phase results revealed a significant increase in Ure in soil variants M and M + S, which were amended with the manure types most abundant on the total nitrogen, 24.79 g·kg^−1^ (M) and 23.93 g·kg^−1^ (M + S), (Table 3). The positive correlation between Ure and AGB dry in the early phase of experiment was in the line with reported correlation of nitrogen mineralization rate with urease activity^83^. The final (24 weeks) values of Ure were comparable in variants amended with enriched manure, M + B, M + S, M + B + S and the control, and Ure and AGB dry correlated negatively but insignificantly at the end of experiment which corresponds with the generally reported contribution of urease to inhibition of crop yield^84^.

## Conclusions

Significant diverse impact of manures co-matured with biochar, elemental sulfur or combination of both on the properties of treated soil was evaluated. This variable effect on dry plant biomass, physico-chemical and biological traits of soil variants was time-dependent, as it was evident from the differences between samples from 12 week-lasting (with barley) and 24 week-lasting (with maize) pot experiment carried in a growth chamber. Co-matured manure with biochar (M + B) showed the highest rate of maturation, its amendment to soil lead to significantly highest soil fertility and barley dry aboveground biomass in the half-time (12 weeks) of experiment, however, the effect vanished after 24 weeks. The highest long-term (24 weeks) contents of total carbon and nitrogen was received for this variant. M + B showed most significant longer-term effect of co-matured manure with biochar on nutrient sequestration and SOM stabilization in soil. Contrarily, after 12 weeks, co-matured manure with biochar and elemental sulfur (M + B + S) impaired some properties to the treated soil (e.g., the lowest pH) which led to short-term carbon sequestration (the highest TC) due to presumed retardation of microbial-mediated transformation of nutrients. This effect of M + B + S manure faded out after 24 weeks as well. Further, there was observed also a specific effect of co-matured manure with only elemental sulfur (M + S). After 12 weeks, its application led to the significantly decreased respiration induced by amino acids (L-alanine, L-arginine). Longer (24 weeks) interaction of manure M + S resulted in basal and all induced respiration values comparable to the control, the lowest basal respiration.

Therefore, we conclude that enriched co-matured manure variants exerted significantly changed properties in comparison to the unenriched manure. However, their application to soil brought only short-term (12 week) beneficial effect on soil fertility and barley plant biomass, compared to single-composed manure. The longer-term (24 weeks) benefit was not significant. We assumed that the prolonged pot experiment with biochar or elemental sulfur enriched manure led to the increased recalcitrancy of soil organic matter (SOM) and retardation of soil nutrient transformation to the plant-available form. We probably should verify our findings in further research, preferably upscaled to the small-scale-plot format.

## Supplementary Information

Supplementary Information.

## Data Availability

The datasets used and/or analysed during the current study are available from the corresponding author on reasonable request.
