# Supplementary material for: Time-dependent impact of co-matured manure with elemental sulfur and biochar on the soil agro-ecological properties and plant biomass
Source: Sci Rep. 2023 Mar 15;13:4327. doi: 10.1038/s41598-023-31348-7 (PMC10017759; doi:10.1038/s41598-023-31348-7)
Supplement: Supplementary file 1 — Supplementary Information. [file 41598_2023_31348_MOESM1_ESM.docx]

**Supplementary materials**

a)
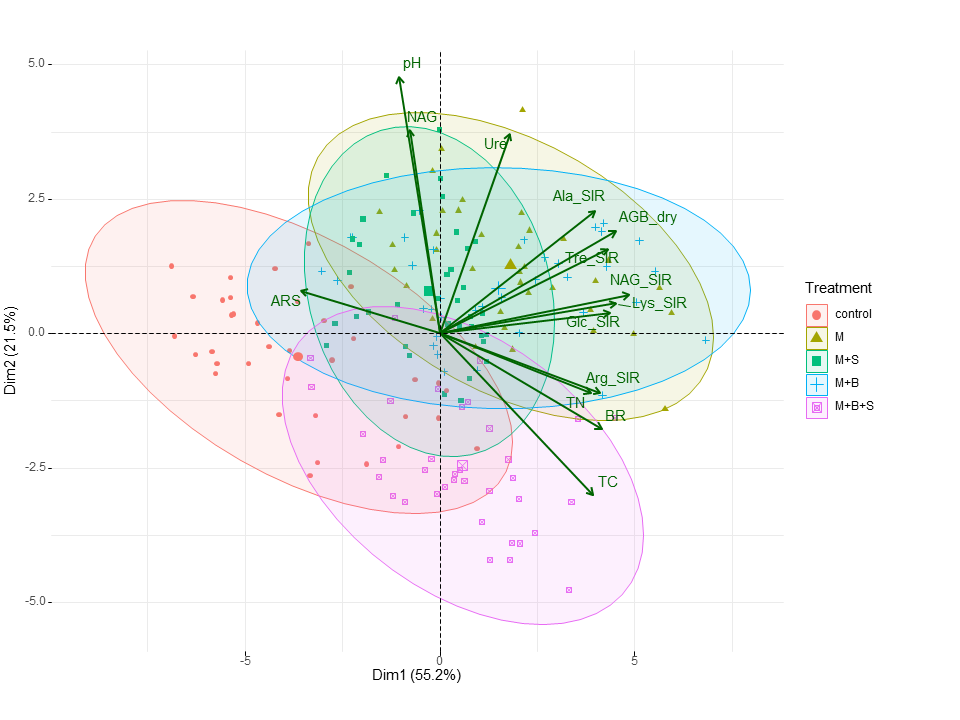


b)


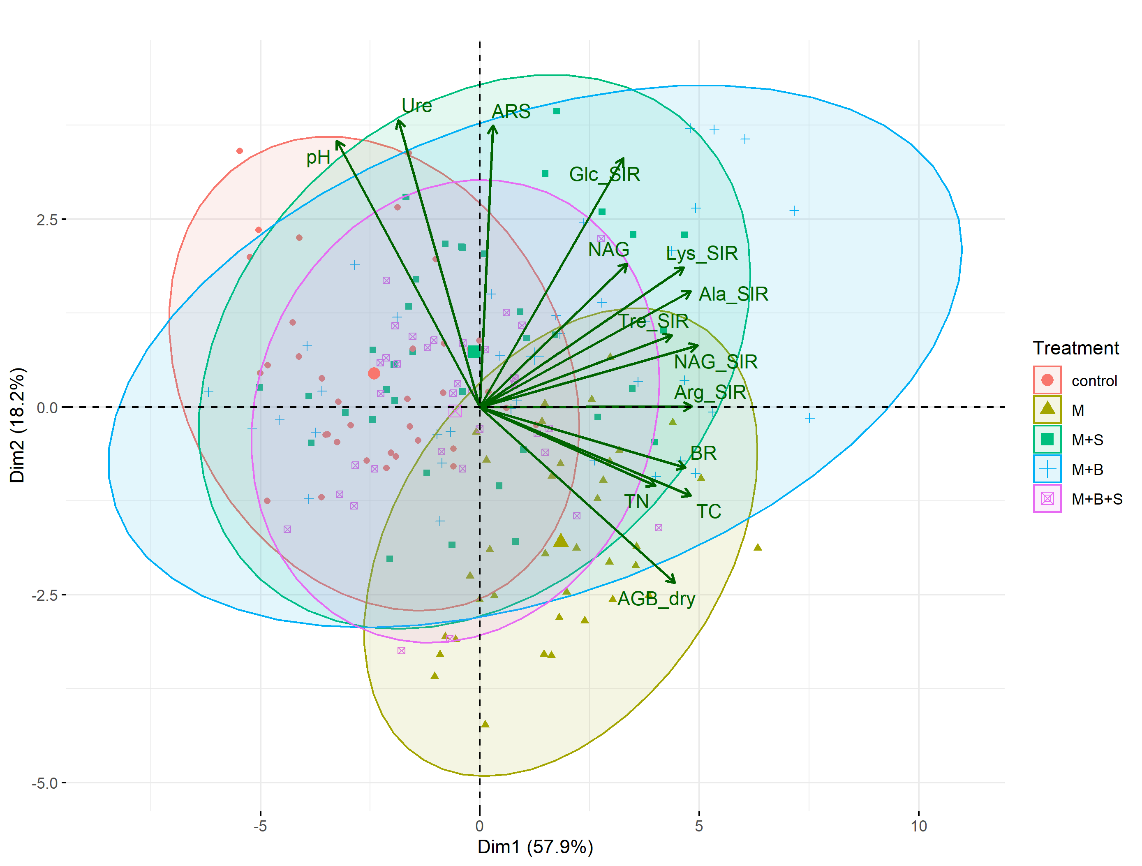


### **Fig. S1. The PCA biplot of plant and soil properties after (a) 12 and (b) 24 weeks of cultivation**

a)


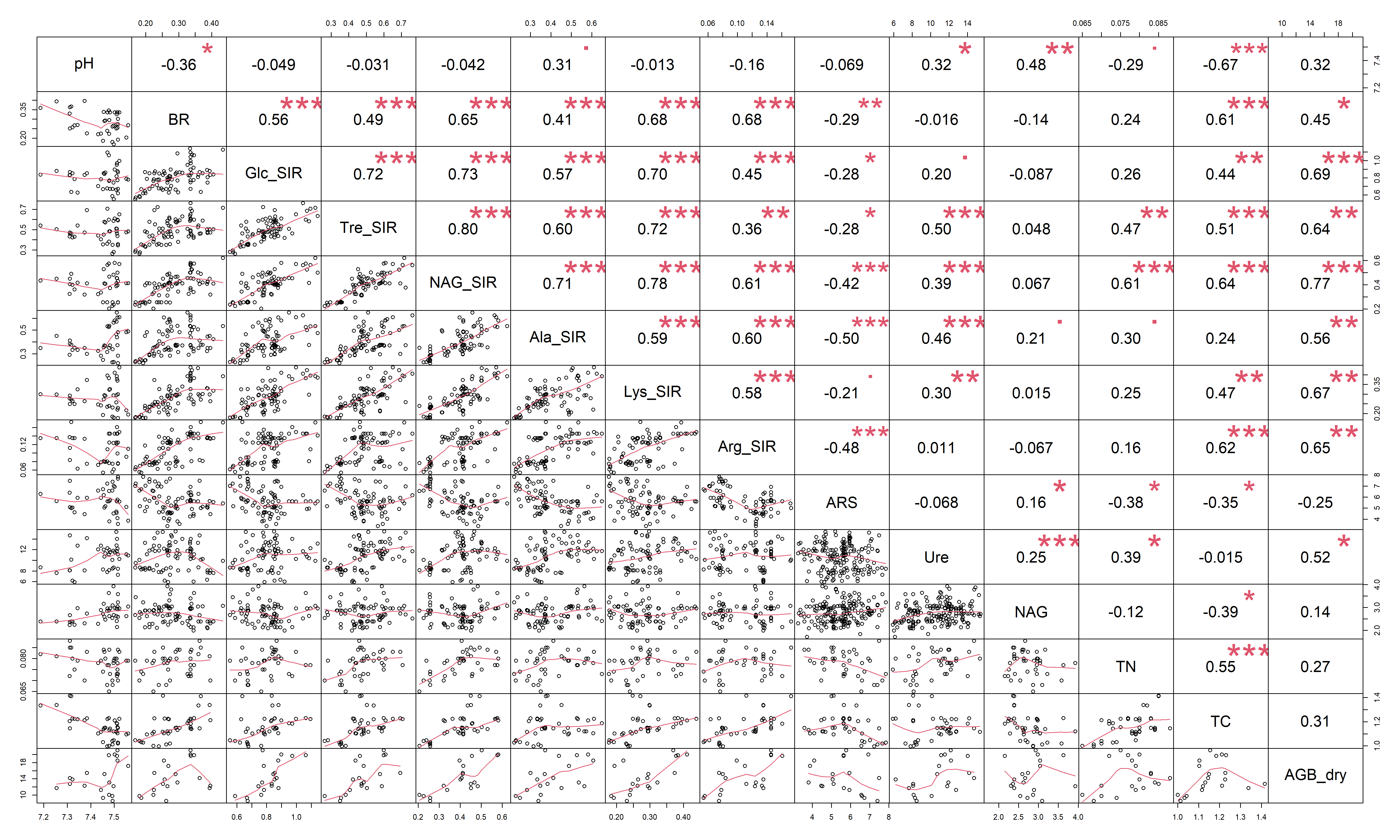


b)


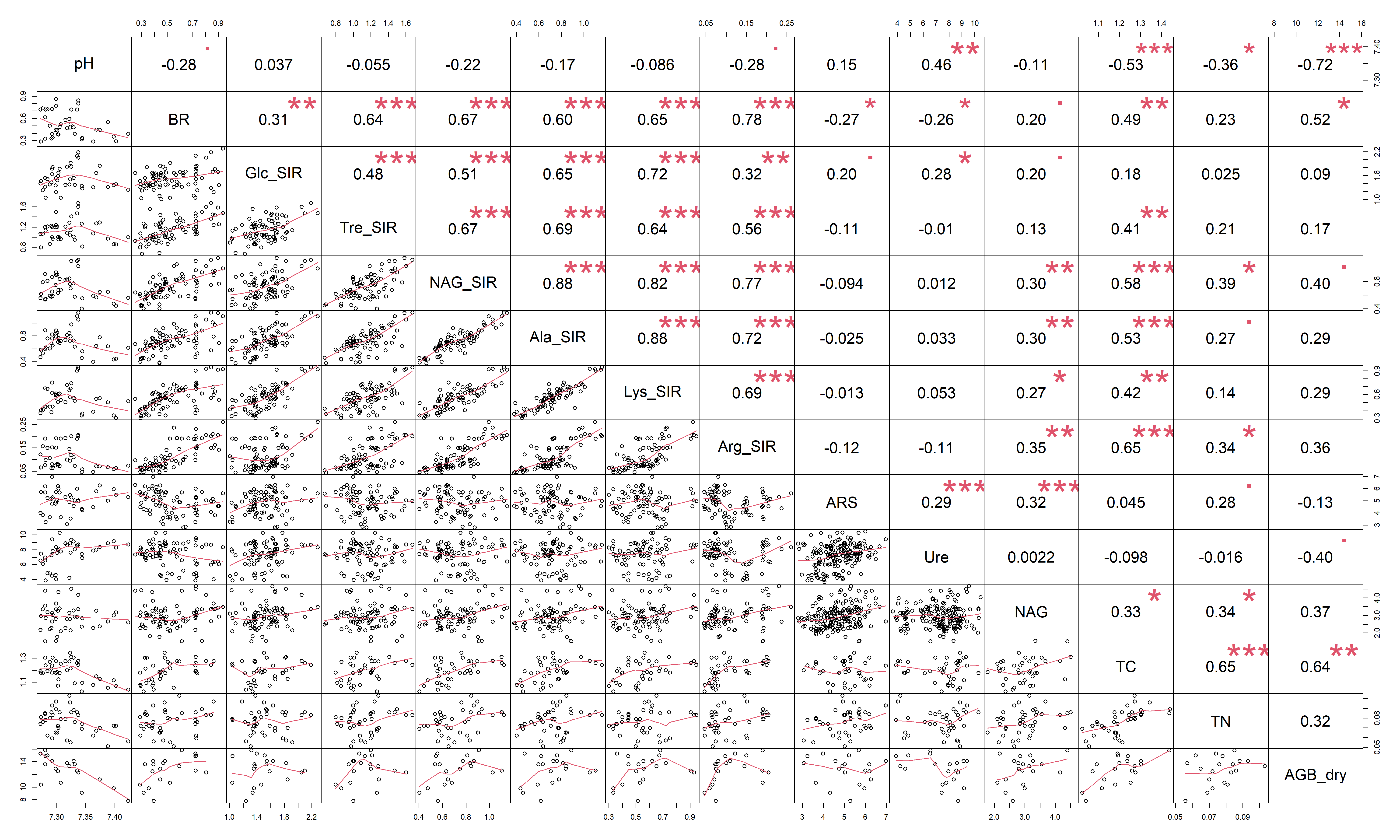


### **Fig. S2. Pearson’s correlation matrix of plant and soil properties after (a) 12 and (b) 24 weeks of cultivation**

*Displayed are correlation coefficients (r), calculated at statistical significance level (p) of: ∙ ≤ 0.1, * ≤ 0.05, ** ≤ 0.01, *** ≤ 0.005.*
